# Maternal awareness and practices regarding vitamin D and their impact on health outcomes in children under five years in Egypt

**DOI:** 10.1038/s41598-026-54282-w

**Published:** 2026-06-17

**Authors:** Seham El-Sayed Saleh, Abdelaziz Hendy, Rasha Kadri Ibrahim, Eman Arafa Badr

**Affiliations:** 1 Pediatric Nursing Department, Faculty of Nursing, Matrouh University, Marsa Matrouh, Egypt; 2 Department of Maternal and Child Health Nursing, College of Nursing, Qassim University, 52571 Buraydah, Saudi Arabia; 3 Pediatric Nursing Department, Faculty of Nursing, Ain Shams University, Cairo, Egypt; 4 Nursing Department, Fatima College of Health Science, Madinat Zayed, Al Dhafra Region, UAE; 5 Pediatric Nursing Department, Faculty of Nursing, Damahur University, Damanhur, Egypt; 6 Pediatric Nursing Department, Faculty of Nursing, Alexandria University, Alexandria, Egypt

**Keywords:** Vitamin D deficiency, Child, Mothers, Dietary supplements, Awareness, Practices, Diseases, Health care, Medical research, Risk factors

## Abstract

Vitamin D is essential for calcium absorption, bone mineralization, and immunological function; nonetheless, vitamin D deficiency (VDD) continues to be a significant health issue in children under five years. In Egypt, VDD is highly prevalent among preschool children, with maternal awareness and nursing practices playing significant roles in its occurrence. This study aimed to identify the relationship between maternal awareness and practices regarding vitamin D and their impact on health outcomes in children under five years. A cross-sectional, correlational, descriptive, and quantitative study was employed. Data collection occurred over eight months throughout three governorates in Egypt, involving 300 mothers and their children under five years of age. Children’s health status outcomes were assessed and linked with their mothers’ awareness and practice scores regarding vitamin D supplementation. It was found that two-thirds of mothers had satisfactory knowledge about vitamin D deficiency, but more than half showed unsatisfactory practice scores. A moderate positive correlation was found between maternal practices and children’s health outcomes as height (r = 0.301, *p* < 0.001), and weight (0.242, *p* < 0.001), as well as between mothers’ practices and knowledge scores (*r* = 0.323, *p* < 0.001). Additionally, maternal practices were significantly influenced by both age and educational level. Maternal practices emerged as a more convincing and coherent associated with child health outcomes than awareness alone. The findings underscore revealed that although over two-thirds of mothers had satisfactory knowledge about VDD, more than half showed unsatisfactory practices. Mothers of children without VDD, and those whose primary teeth erupted before one year demonstrated significantly higher practice scores. A moderate positive correlation was found between maternal practices and children’s health outcomes (weight, and height), as well as between mothers’ practices and awareness scores. Additionally, maternal practices were significantly influenced by both age and educational level.

## Introduction

Vitamin D is essential for calcium and phosphorus absorption, bone mineralization, and maintenance of skeletal health^1^. Deficiency or resistance to vitamin D may lead to hypocalcemia and hypophosphatemia and remains one of the most common undiagnosed medical conditions worldwide^2^. Vitamin D deficiency (VDD) is widely recognized as a common nutritional deficiency affecting different age groups and both sexes across countries^2–5^. In Egypt, VDD is highly prevalent among preschool children, reaching 54.4%, with maternal education and breastfeeding duration significantly influencing children’s serum vitamin D levels^2^. During childhood, VDD is associated with growth retardation and nutritional rickets^6^. Recent evidence also suggests that low vitamin D levels may contribute to extra skeletal disorders through immunomodulatory and anti-inflammatory mechanisms^7,8^, including allergic diseases^9^, respiratory illnesses^10^, and obesity in children^11^. Long-term consequences may include impaired peak bone mass and increased risk of osteoporotic fractures. VDD has also been linked to several chronic diseases, including autoimmune, infectious, cardiovascular, malignant, and neurodegenerative conditions^2–6^.

The Recommended Dietary Allowance (RDA) for vitamin D is 400 IU daily for newborns up to 12 months and 600 IU for children aged 1 to 18 years. Infants can obtain sufficient vitamin D by 30 min of sunlight exposure per week while wearing only a diaper, or 2 h per week when fully clothed and without a hat^12–14^. The American Academy of Pediatrics (AAP) recommends that children under 6 months be shielded from direct sunlight exposure due to potential skin cancer risks later in life, advocating for a natural diet or vitamin D supplementation as preferable alternatives^12,13^. This supplementation should persist until infants consume over 1000 ml per day of vitamin D-fortified formula. Given that the majority of infant formulae provide a minimum of 400 IU/L of vitamin D, formula-fed infants may require vitamin D supplementation unless their intake exceeds 1000 mL per day^14,15^.

Parents of VDD children need pediatric nurses to advise them of its risks. As mothers, they must learn about VDD to prevent it. Nurses should emphasize sun exposure and Vitamin D sources to mothers^16^. Vitamin D is one of the fat-soluble vitamins. The pediatric nurse should advise mothers to eat vitamin D-rich foods, including sardines, herring, tuna, mackerel, salmon, cod liver oil, egg yolks, shiitake mushrooms, and organ meats. 90% of vitamin D is synthesised by the skin after UVB exposure^14,15^.

The children’s nutritional contents are influenced basically by the awareness of mothers, which is considered healthy or not, which is considered an important responsibility of the pediatric nurs^17^. The health of children, especially the infants’, is strongly associated with their mothers’ awareness, as they are the primary source of care. Thus, it was urgently crucial to enrich the mothers’ knowledge and understanding of the possible leading causes for decreasing the levels of such an important Vitamin^18^. So, mothers are primarily responsible for the children’s nutritional needs^17^. Therefore, this study aims to identify the relationship between maternal awareness about vitamin D and health status outcomes of their children under 5 years old.

Vitamin D deficiency is a global health problem in children and is considered an epidemic worldwide. It is a serious public health problem in both developed and developing countries, including the Middle East and North African region, which includes Egypt, which has a very high rate of VDD which reaching 81% among various age groups^19–21^.

Assessment of mothers’ awareness regarding VDD helps to close the gap between the mothers’ knowledge and practices and will correct the misunderstood concepts regarding their children’s health. Defining the highly important role which done by Vitamin D plays in children’s health. It is necessary to examine the mothers’ knowledge and practices with children regarding Vit D and identify where the gap could be in their knowledge background^2^. So, it was very important to be aware of the relationship between maternal awareness about vitamin D for children under five years and their health status outcomes to maintain and promote health among their children. This study aimed to identify the relationship between maternal awareness and practices regarding vitamin d and their impact on health outcomes in children under five years.

### Hypothesis

There is a statistically significant positive correlation between maternal awareness and practices and the health outcomes of children under five years (BMI, weight, height, and absence of vitamin D deficiency manifestations).

### Operational definitions

#### Health status outcomes

This study includes manifestations of VDD, the child’s physical growth (height, weight, and Body Mass Index).

## Methods

### Research design, setting, and sample

A cross-sectional, correlational, descriptive, and quantitative methodology was employed to conduct this study. A cross-sectional correlational design was selected as it allows for the assessment of relationships between maternal awareness, practices, and child health outcomes at a single point in time without manipulating variables. The report adheres to the requirements established by Strengthening the Reporting of Observational Studies in Epidemiology (STROBE), conducted from January to August 2024.

### Study setting

This study was conducted at three different governorates in Egypt by collecting data from mothers in the Outpatient Departments of the children’s hospitals at Smouha Children^'^s University Hospital (SCUH) in Alexandria governorate, Matruh General Hospital and Specialized Children’s Hospital in Matruh governorate, and Damanhur National Medical Institute in El-Behara Governorate in Egypt.

### Sample

A convenience sampling technique was used to recruit 300 mothers and their children under five years of age from the outpatient departments of the selected hospitals. Participants were approached during their routine visits to the pediatric outpatient clinics, and those who met the eligibility criteria were invited to participate voluntarily in the study after explaining the purpose of the research.

Inclusion criteria included: mothers having children under five years of age, mothers who were willing to participate, and children who were free from neurological and chronic diseases. Exclusion criteria included: children with diagnosed chronic illnesses or congenital disorders that could affect growth and nutritional status, and mothers who refused to participate or were unable to complete the interview. All participants provided informed consent prior to participation.

The sample study was estimated based on the Epi-Info 7 program, based on the following parameters: Children’s size = 450, Expected frequency = 50%, Margin of error = 5%, Confidence level = 95%, Minimum sample size was 208.

## Data collection tools

### Tool 1: characteristics of mothers

Age, residence, level of education, occupation, marital status, and number of children less than five years were attached to the instrument.

### Tool 2: mothers’ awareness regarding vitamin D

Was developed by the researcher based on reviewing relevant literature^2,22^ to identify mothers’ knowledge and their practices regarding vitamin D supplementation. It included three parts:

### Part I: mothers’ knowledge regarding vitamin D supplementation

This section consisted of 18 multiple-choice questions assessing mothers’ knowledge about vitamin D, including its importance, sources, appropriate age for initiation of supplementation, frequency and dosage, methods of sun exposure, dietary sources, and sources of information. Each correct answer was scored as one point, while incorrect or “don’t know” responses were scored as zero. Total scores were summed and converted into percentages. Knowledge levels were categorized as satisfactory (≥ 70%) and unsatisfactory (< 70%) based on the classification adopted from Bassam and Abd-Elmageed^22^.

### Part II: mothers’ practices regarding vitamin D supplementation

This section included 21 items assessing mothers’ practices related to vitamin D, including supplementation (6 items), sun exposure (7 items), and provision of vitamin D-rich foods (8 items). Each correctly performed practice was scored as one point, and incorrect or not performed practices were scored as zero. Total practice scores were summed and converted into percentages. Practice levels were categorized as satisfactory (≥ 70%) and unsatisfactory (< 70%) based on the classification adopted from Bassam and Abd-Elmageed^22^.

### Tool 3: the health status outcomes of children under five years

It was developed by researchers guided by Liang et al.^23^ to identify the children’s health status and comprises three parts:

The selected physical signs and medical history items were based on commonly reported clinical manifestations and complications of vitamin D deficiency in children, as identified in previous studies^18,19,22,23^. These indicators, including delayed motor development, delayed dentition, bone deformities, and recurrent illnesses, are widely used as clinical proxies for vitamin D deficiency, particularly in settings where biochemical assessment is not feasible.

### Part I: manifestations of VDD

Every child was examined and asked the mothers for common signs of vitamin D deficiencies such as delayed closure of fontanels, delayed sitting and walking, delayed dentition (no teeth by 9 months, no molars by 14 months), muscles weakness and cramping, bow leg, knock knee and bone fracture.

### Part II: child’s physical growth

Contained measurement of child’s weight, length/ height, and Body mass index (BMI = weight (kg) / height (m^2^).

### Part III: previous medical and surgical history

Included questions to mothers about the child’s health, such as exposure to any disease, i.e., pneumonia, previous hospitalization, cause of hospitalization, duration, and previous surgery. Each item will be scored with one point for No, and zero points for Yes in case of presence. All responses were summed up. A higher score will indicate a good health status. Children’s health status will be categorized into: Good health status ≥ 70%, Moderate health status < 70% to ≥ 50%, and Poor health status < 50%. Children’s characteristics were added to this instrument and involved the child’s age, gender, and birth order. After the development of study tools by the researchers, they were tested for their content validity by five experts in the pediatric nursing field, and necessary modifications were made.

The study instruments were developed by the researchers after reviewing relevant literature and previously used instruments. To ensure content validity, the tools were submitted to a jury of five experts in pediatric nursing who evaluated the items for relevance, clarity, comprehensiveness, wording, and applicability to the study objectives. Based on their feedback, minor modifications were made to improve the clarity and appropriateness of several items.

The tools were then pilot tested on 10% of the sample (*n* = 30 mothers and their children) to assess feasibility, clarity, and time required for completion. Participants included in the pilot study were excluded from the final analysis. Internal consistency reliability was assessed using Cronbach’s alpha, which showed good reliability for the awareness tool (α = 0.81) and practice tool (α = 0.84).

### Data collection procedures

An official letter was obtained from the Faculty of Nursing, Alexandria University, and submitted to the administrative authorities of the selected study settings to facilitate data collection. Approval to conduct the study was granted after explaining the study objectives. Data were collected through individual face-to-face interviews with each mother in a private setting to ensure confidentiality. Information regarding maternal and child characteristics, as well as mothers’ knowledge and practices related to vitamin D, was obtained using the previously described data collection tools.

Children’s health status was assessed through clinical observation for manifestations of vitamin D deficiency, in addition to measuring weight and length/height to calculate body mass index (BMI). Furthermore, mothers were asked about their children’s medical and surgical history. Data collection was carried out over a period of eight months.

### Pilot study

A pilot study was conducted on 10% of the total sample (*n* = 30 mothers and their children under five years) to evaluate the clarity, feasibility, and applicability of the data collection tools. The pilot study also aimed to estimate the time required for data collection and identify any potential difficulties during implementation. Based on the findings of the pilot study, minor modifications were made to improve the clarity and consistency of the questionnaire items. The participants included in the pilot study were excluded from the final sample to avoid bias.

### Ethical considerations

The Faculty of Nursing at Alexandria University gave the study [AU-20–4-182], IRB00013620 (9/19/2025] ethical approval. The study followed the rules set out in the Declaration of Helsinki. Everyone who took part was given information about their rights and the rules for protecting their data. The poll does not collect personal information, and all replies will be kept safe for research purposes only. All participants in the study gave their informed consent. All procedures were carried out in accordance with relevant rules and regulations.

### Statistical analysis

Data were analyzed using the Statistical Package for the Social Sciences (SPSS, version 26) and Python statistical libraries. Descriptive statistics (mean, standard deviation, and frequency distributions) were calculated for all study variables. The assumption of normality was evaluated using the Shapiro–Wilk test and visual inspection of histograms and Q–Q plots. Variables with *p* > 0.05 in the Shapiro–Wilk test and approximately symmetric histograms were considered to meet the normal distribution criteria.

Independent samples *t*-tests and one-way analysis of variance (ANOVA) were used to compare mothers’ total awareness scores across categories of children’s health outcomes, including VDD diagnosis, timing of first appearance of milky teeth, previous hospitalization, and BMI category. When significant differences were found, effect sizes were calculated using Cohen’s *d* for two-category outcomes and eta-squared (η^2^) for three or more categories to assess the magnitude of the differences.

Pearson’s product–moment correlation coefficients (*r*) were computed to assess the association between mothers’ total awareness scores and continuous child health indicators: body mass index (BMI), height, weight, and times of hospitalization. Correlation coefficients were presented alongside their corresponding *p*-values to indicate statistical significance. A significance level of *p* < 0.05 was adopted for all inferential tests. Effect sizes were interpreted according to Cohen’s conventions: small (|*d*|= 0.2), medium (|*d*|= 0.5), and large (|*d*|= 0.8) for *d*, and small (η^2^ = 0.01), medium (η^2^ = 0.06), and large (η^2^ = 0.14) for η^2^.

## Results

The study included 300 children with a mean age of 1.84 ± 1.34 years, most of whom were aged 1–3 years (37.7%) and male (54.3%). The largest proportion were third-born (32.3%). Common health problems included weight gain (39.0%) and malnutrition (29.0%). Most children (79.3%) experienced delayed eruption of primary teeth, and 85.3% had a history of hospitalization. Vitamin D deficiency was reported in 56.3% of children, while only 29.7% received treatment. The majority were underweight (89.3%), with a mean BMI of 13.27 ± 4.29. Mean height and weight were 85.58 ± 10.97 cm and 9.71 ± 3.67 kg, respectively, see Table 1.

**Table 1:** Characteristics of studied children (*n* = 300).

Variable	Category	*n*	%
Child age	< 1 year	99	33.0
	1 to less than 3 years	113	37.7
	3 to less than 5 years	88	29.3
	Mean (SD). 1.84 (1.34)		
Gender	Male	163	54.3
	Female	137	45.7
Order of the child	The third	97	32.3
	The second	73	24.3
	The fourth	68	22.7
	The first	41	13.7
	The fifth	21	7.0
Health problems	Weight gain	117	39.0
	Malnutrition	87	29.0
	Intestinal health problems that affect the absorption of vitamin D	55	18.3
	Kidney disease	31	10.3
	Liver disease	10	3.3
First appear Milky teeth	After one year	238	79.3
	Before one year	62	20.7
Previous hospitalization	Yes	256	85.3
	No	44	14.7
Diagnosed with VDD	Yes	169	56.3
	I don’t know	88	29.3
	No	43	14.3
Treatment for Vit D deficiency	No	172	57.3
	Yes	89	29.7
	I don’t know	39	13.0
Injection treatment of vitamin D	No	268	89.3
	Yes	32	10.7
BMI	Underweight	268	89.3
	Ideal	30	10.0
	Overweight	2	0.7
	Mean (SD). 13.27 (4.29)		
Child weight	Mean (SD). 9.71 (3.67)		
Child height	Mean (SD). 85.58 (10.97)		

The study included 300 mothers with a mean age of 25.64 ± 9.36 years, most of whom were under 20 years (41.3%) and from rural areas (69.0%). The majority lived in private housing (78.3%) and were housewives (55.3%), with 78.7% being married. Educational levels varied, with 39.3% having secondary education. About 43.7% had four children, and 66.7% had one child under five. Over half demonstrated unsatisfactory practices (52.7%), while 69.7% had satisfactory awareness levels, see Table 2.

**Table 2:** Characteristics of studied mothers (*n* = 300).

Items	Categories	*n*	%
Mother. age	< 20 years	124	41.3
	20 to less than 35 years	105	35.0
	> 35 years	71	23.7
	Mean (SD). 25.64 (9.36)		
Residence	Rural	207	69.0
	Urban	93	31.0
Housing	Private	235	78.3
	Rent	65	21.7
Educational	Secondary education	118	39.3
	Basic education	108	36.0
	Illiterate	52	17.3
	University	22	7.3
Occupation	Housewife	166	55.3
	Employee	134	44.7
Marital status	Married	236	78.7
	Widow	44	14.7
	Divorced	20	6.7
The Number of children	Four	131	43.7
	Five	81	27.0
	Three	48	16.0
	Two	40	13.3
Number of children under 5	One	200	66.7
	Two	78	26.0
	Three	22	7.3
Total knowledge	Satisfactory	209	69.7
	Unsatisfactory	91	30.3
	Mean (SD). 30.91 (7.23)		
Total practice	Unsatisfactory	158	52.7
	Satisfactory	142	47.3
	Mean (SD). 33.27 (5.09)		

Table 3 compares the mother**s’** total practice scores with their children’s outcomes. Mothers of children without VDD exhibited significantly higher practice scores (M = 36.42, SD = 2.60) compared to those with deficient children (M = 32.92, SD = 6.42), t = 5.519, *p* < 0.001, indicating a moderate effect size (0.597). Mothers of children whose primary teeth erupted before one year exhibited elevated practice scores (M = 34.62, SD = 4.00), in contrast to those whose children’s teeth erupted after one year (M = 28.11, SD = 8.63), t = 5.779, *p* < 0.001, indicating a substantial effect size (1.23). There were no significant differences in practice scores based on previous hospitalization (*p* = 0.09) or BMI category (*p* = 0.45), with both exhibiting negligible to small effect sizes.

**Table 3:** Comparison of mothers’ total practice scores with their children’s outcomes.

Outcome	Category	Mothers’ total practice scores				
		Mean	SD	Test statistics (t/F)	*p*-value	Effect size
Vit D deficiency diagnosis	No	36.42	2.6	5.519	0.000	0.597
	Yes	32.92	6.42			
First appearance of milky teeth timing	After one year	28.11	8.63	5.779	0.000	1.23
	Before one year	34.62	4.0			
Previous hospitalization	No	31.66	6.87	1.727	0.09	0.322
	Yes	33.55	5.69			
BMI category	Under weight	33.14	5.94	0.80	0.45	0.005
	Overweight	32.0	4.24			
	Ideal	36.79	3.8			

Table 4 shows how mothers’ total awareness scores compare to their children’s outcomes. There was no significant difference in awareness scores based on VDD diagnosis (*p* = 0.577) or BMI category (*p* = 0.787), and both showed very small effect sizes. Nevertheless, the timing of the emergence of the first molars was significantly correlated with maternal awareness; mothers whose children’s teeth erupted before one year exhibited higher mean awareness scores (M = 20.35, SD = 4.90) in contrast to those whose children’s teeth erupted after one year (M = 18.40, SD = 6.13), t = 2.313, *p* = 0.023, indicating a small to moderate effect size (0.376). Prior hospitalization did not exhibit a significant correlation with awareness scores (*p* = 0.247).

**Table 4:** Comparison of mothers’ total awareness scores with their children’s outcomes.

Outcome	Category	Mothers’ total awareness scores				
		Mean	SD	Test statistics (t/F)	*p*-value	Effect size
Vit D deficiency diagnosis	No	19.76	5.14	0.558	0.577	0.065
	Yes	20.09	5.31			
First appearance of milky teeth timing	Before one year	20.35	4.9	2.313	0.023	0.376
	After one year	18.4	6.13			
Previous hospitalization	No	19.02	5.75	1.171	0.247	− 0.207
	Yes	20.11	5.13			
BMI category	Underweight	19.93	5.26	0.239	0.787	0.002
	Ideal	19.9	5.18			
	Overweight	22.5	0.71			

Among the 300 children, the most commonly reported VDD-related symptoms were overweight (21.7%), fractures without injury (21.0%), colds (21.0%), asthma (21.0%), and malnutrition (21.0%). Other manifestations included gastrointestinal infections and convulsions (20.7% each), muscle weakness (18.7%), and skeletal deformities (18.0%). Additionally, abnormal tooth development (16.7%) and delayed teething (16.0%) were observed. Approximately 19% of children exhibited delayed fontanelle closure and delayed motor development, see Table 5.

**Table 5:** Symptoms of VDD among studied children (*n* = 300).

Symptoms	Categories	n	%
Bone weakness and pain, muscle weakness	No	244	81.3
	Yes	56	18.7
Muscle cramps	No	242	80.7
	Yes	58	19.3
Delay closure of fontanelles	No	242	80.7
	Yes	58	19.3
Delayed sitting and crawling	No	242	80.7
	Yes	58	19.3
Delayed start of walking	No	242	80.7
	Yes	58	19.3
Bowing of the legs or knock knees	No	250	83.3
	Yes	50	16.7
Abnormal tooth development	No	250	83.3
	Yes	50	16.7
Delayed teething	No	252	84.0
	Yes	48	16.0
Abnormal curvature of the spine and bowing of the legs or knock knees	No	246	82.0
	Yes	54	18.0
Fractures without actual injury	No	237	79.0
	Yes	63	21.0
Colds	No	237	79.0
	Yes	63	21.0
Asthma	No	237	79.0
	Yes	63	21.0
Malnutrition	No	237	79.0
	Yes	63	21.0
Overweight	No	235	78.3
	Yes	65	21.7
Gastrointestinal infections	No	238	79.3
	Yes	62	20.7
Convulsions	No	238	79.3
	Yes	62	20.7
Pneumonia	No	238	79.3
	Yes	62	20.7

Table 6 shows how children’s anthropometric and hospitalization variables are related to their mothers’ total awareness and practicescores. Total awareness scores did not exhibit a significant difference. Conversely, There were weak to moderate positive correlations between total practice and height (r = 0.301, *p* < 0 0.001) and weight (r = 0.242, *p* < 0.001). There was also a weak but significant negative correlation between total practice and times of hospitalization (r =  − 0.145, *p* = 0.012). BMI exhibited no significant correlation (*p* = 0.091).

**Table 6:** Correlation between BMI, height, weight, time of hospitalization with mothers’ awareness, and practice.

Variable	Total awareness r. (*p*)	Total practice r. (*p*)
BMI	0.049 (*p* = 0.397)	0.098 (*p* = 0.091)
Height	0.076 (*p* = 0.187)	0.301 (*p* = 0.00)
Weight	0.108 (*p* = 0.062)	0.242 (*p* = 0.00)
Times of hospitalization	− 0.085 (*p* = 0.14)	− 0.145 (*p* = 0.012)

**Fig. 1:**
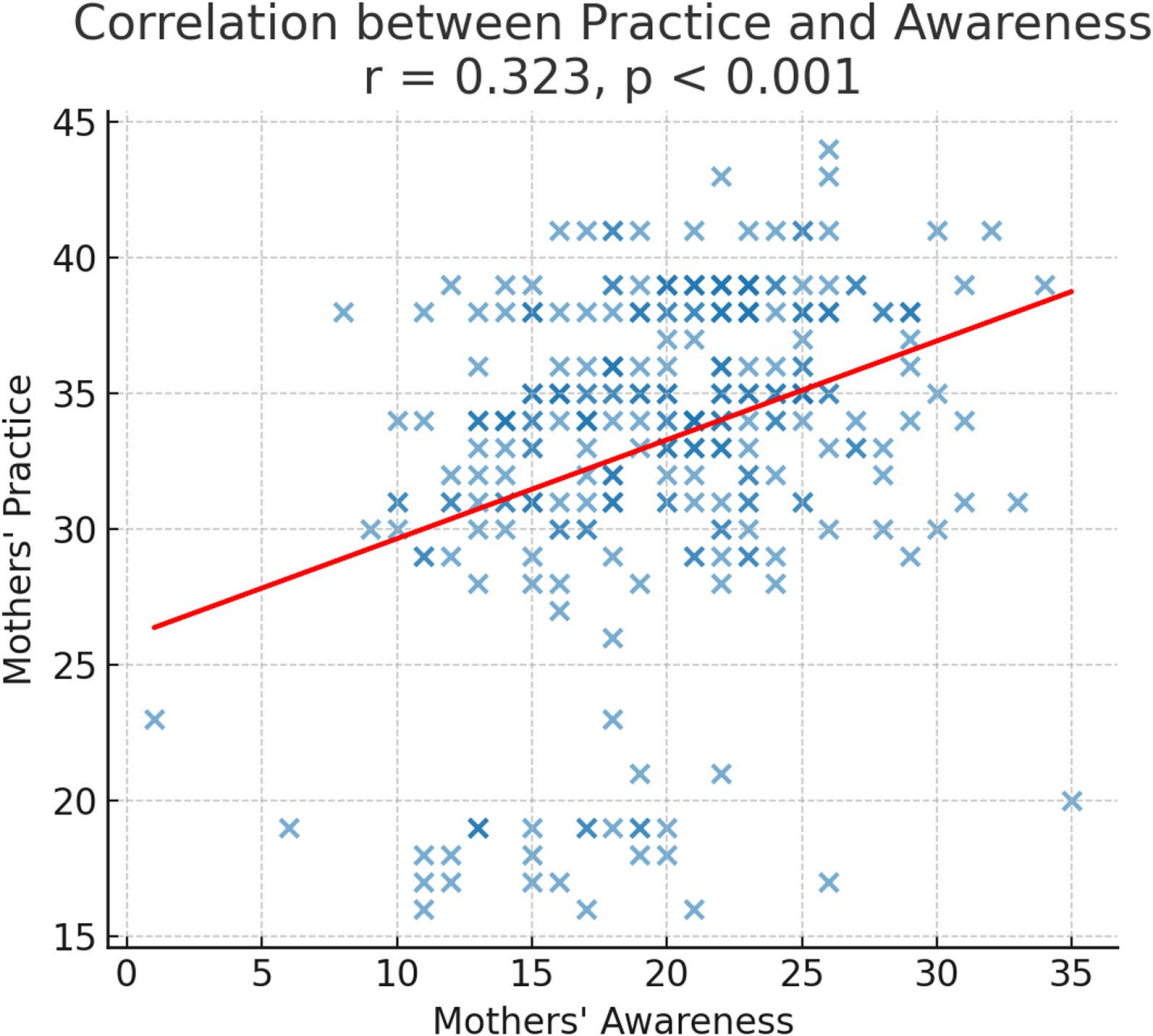
Correlation between mothers’ practice and awareness.

Figure 1 illustrates a positive correlation between mothers’ awareness and practice scores (*r* = 0.323, *p* < 0.001).

Table 7 compares mothers’ total practice scores with their characteristics. Maternal age showed a statistically significant association with practice scores (*p* = 0.007), with mothers aged 20 to < 35 years having the highest mean score (M = 34.53, SD = 4.04) compared to those under 20 years (M = 32.1, SD = 6.63) and over 35 years (M = 33.45, SD = 6.51), though effect size was small (0.032). Mothers with a university degree had the highest mean score (M = 36.0, SD = 6.06), while mothers without a university degree had the lowest mean score (M = 31.73, SD = 5.28), although the effect size was small (0.028). Educational level was also significant (*p* = 0.037). While employees and rural residents had slightly higher mean practice scores than housewives and urban residents, there were no significant associations found for housing type, occupation, or residence (*p* > 0.05).

**Table 7:** Comparison of mothers’ total practice scores with their characteristics.

Mother characteristic	Category	Total practice scores				
		Mean	SD	Test statistics (t/F)	*p*-value	Effect size
Mother. Age	> 35 years	33.45	6.51	4.987	0.007	0.032
	< 20 years	32.1	6.63			
	20 to < 35 years	34.53	4.04			
Housing	Private	33.27	5.85	0.005	0.995	-0.001
	Rent	33.28	6.11			
Educational	Secondary education	33.23	4.34	2.863	0.037	0.028
	Basic education	33.51	7.31			
	University	36.0	6.06			
	Illiterate	31.73	5.28			
Occupation	Employee	33.84	4.41	1.575	0.116	0.175
	Housewife	32.81	6.85			
Residence	Rural	33.64	5.59	1.511	0.132	0.2
	Urban	32.46	6.5			

## Discussion

Childhood is a critical period for establishing suitable patterns of nutritional behaviors. These behaviors are directly affected by the mothers’ beliefs about the nutritional quality of their children. Improving mothers’ nutritional knowledge can cause changes in their children’s nutritional behavior^24^.Therefore, the existing study aimed to identify the relationship between maternal awareness about vitamin D for children under five years and their health status outcomes.

The current findings indicated that a significant percentage of children with VDD were underweight, with a mean BMI of 13.27 (SD = 4.29). This outcome underscores the strong correlation between vitamin D levels and the dietary growth results of children. Furthermore, it substantiates global evidence that Vitamin D serves not only as a nutrient for bone health but also as an indicator of overall child nutrition and growth potential. Multiple mechanisms may elucidate this connection. Underweight children frequently exhibit inadequate nutritional consumption, lacking not just in calories and protein but also in vital micronutrients, such as vitamin D, which exacerbates growth retardation^25^. Secondly, VDD has been associated with heightened susceptibility to infections, including respiratory tract diseases, which may exacerbate nutritional status due to recurrent illness and diminished appetite^26^.

Children’s health is shaped by their parents’ knowledge and attitudes, which impact their overall well-being and dietary habits. Therefore, parental education is essential in any illness prevention plan. Prior research conducted in several nations indicated that knowledge regarding vitamin D was restricted^27^. The results demonstrate that over two-thirds of the mothers attained a satisfactory total knowledge score on vitamin D and its supplementation, indicating a comprehensive grasp of vitamin D deficiency (VDD). This indicates that women had prior exposure to pertinent information that enhanced their awareness. The standard deviation of 7.23 indicates significant variability in knowledge levels among individuals, suggesting that while the majority performed adequately, some displayed serious deficiencies.

The researcher’s perspective of the knowledge level concerning VDD may stem from the educational responsibilities of healthcare workers in instructing and teaching mothers about the significance of nutrition and vitamins for optimal infant development. The elevated educational attainment of mothers favorably influences their understanding of their children’s needs, specifically regarding vitamin D. These findings are supported by a study conducted in Egypt by Soliman et al.^28^, Also, aligns with research conducted among Saudi Arabian mothers^22,29^, which indicated that a comparable sample possessed substantial knowledge regarding the advantages of vitamin D in the treatment of bone diseases and rickets, as well as in the maintenance of calcium and phosphate levels, bone and dental health, and the enhancement of immunity and muscle strength. Conversely, a study conducted at four health care centers in Egypt revealed that merely 8.8% of Egyptian mothers have an adequate understanding of vitamin D and its inadequacy^30^.

Regarding the children’s outcomes in relation to their mothers’ overall knowledge, no significant differences were observed in mothers’ total knowledge scores depending on VDD diagnosis or BMI category, with both exhibiting minimal impact sizes. The results suggest that children’s outcomes, overall, did not significantly vary based on their mothers’ comprehensive knowledge scores concerning VDD, except in certain developmental areas. No statistically significant differences in maternal knowledge were detected about the child’s diagnosis of VDD or across BMI categories, both exhibiting minimal effect sizes. This indicates that maternal knowledge alone may not significantly impact these health parameters and that various other factors, including diet, genetic predisposition, and environmental exposure, may play a more critical role in determining the child’s physical, mental, and social health status. These rationales are supported by a study conducted in Pakistan^31^.

However, the timing of the eruption of the first molars showed a significant association with maternal knowledge. Mothers whose children’s first molars erupted before one year of age had higher awareness scores compared to those whose children experienced eruption after one year. This significant difference, with an effect size of 0.376, indicates a small to moderate relationship, suggesting that timely dental development might reflect greater maternal attention to nutrition, supplementation, or preventive health measures. In contrast, prior hospitalization of the child was not significantly related to maternal knowledge scores, implying that hospitalization events are more influenced by acute or unrelated conditions rather than chronic nutritional awareness. Overall, the results highlight that while maternal knowledge about VDD may not universally predict all child health outcomes, it could be more closely tied to certain developmental milestones, such as dental eruption timing^32,33^.

The present investigation indicated that over fifty percent of mothers exhibited inadequate practices for VDD. This outcome may be attributed to the fact that, despite Egypt’s abundant sunshine year-round, societal limitations and traditional traditions of excessively covering infants to maintain warmth hinder neonates from obtaining sufficient sunlight exposure. Additionally, numerous women are veiled due to cultural restrictions, resulting in insufficient exposure to sunshine. This outcome is consistent with prior investigations^28,34^. They determined that fewer than two-thirds of the mothers exhibited inadequate practices for VDD. Conversely, Alharbi et al. presented contradictory findings, revealing that over half of the mothers (56.4%) exhibited a high level of practice^35^.

Concerning the correlation between maternal habits and the health results of their children. The results indicate a distinct correlation between maternal habits and specific child health markers, notably vitamin D levels and the time of primary tooth eruption. Mothers of children without VDD exhibited substantially higher practice scores compared to those with deficiency; effect size = 0.597. The moderate impact size indicates that improved maternal practices, including encouraging sun exposure, incorporating dietary sources high in vitamin D, and providing supplementation when required, may significantly contribute to the prevention of insufficiency^36^.

VDD in early childhood has been associated with impaired bone development, rickets, and potential delays in tooth eruption, which may further substantiate the correlation between practice scores and dental milestones. The current study validated this observation, indicating that mothers of infants whose primary teeth emerged before one year exhibited superior practice scores. This conclusion may be elucidated by the notion that elevated maternal practice ratings likely indicate superior feeding behaviors (including breastfeeding and complementary feeding practices), hygiene, preventive care, and other elements recognized to foster normal dental development. Consistent with the findings of Xavier et al.^37^, early tooth eruption may be influenced by proper nutrition, sufficient vitamin D levels, and overall health habits.

The lack of substantial differences in practice scores related to hospitalization history or BMI category, as indicated in our study, implies that although these factors are crucial for child health, they may not be directly affected by the maternal practices evaluated in this study, or the practices assessed were not closely associated with acute illness episodes or weight status. This finding aligns with research suggesting that BMI is complex, affected by genetic, metabolic, and environmental factors, rather than exclusively maternal practices^38^.

Examining the relationship among BMI, height, weight, duration of hospitalization, and the overall understanding and practices of mothers. The overall knowledge scores did not demonstrate a significant association with any of the examined variables. In contrast, a moderate positive connection existed between total practice and both height and weight. A weak yet substantial negative connection existed between total practice and the frequency of hospitalizations. BMI demonstrated no substantial correlation. These findings underscore a significant disparity between knowledge and practice about children’s health outcomes. The lack of a substantial link between total knowledge awareness scores and the examined factors indicates that maternal knowledge may not directly influence quantifiable health indicators such as growth or hospitalization. This aligns with prior research by Glanz et al.^39^, which demonstrates that knowledge alone does not ensure behavioral change without the integration of effective skills, resources, and motivation.

Conversely, practice scores demonstrated significant correlations with kid anthropometrics. The moderate positive correlations between height and weight suggest that superior maternal practices are associated with enhanced growth outcomes. This discovery highlights the significance of tangible caregiver actions, like feeding methods, hygiene, and regular health care, in fostering healthy infant growth. These results correspond with WHO and UNICEF recommendations that highlight maternal and familial practices as essential factors influencing child growth and survival^38,40^.

Notably, practice had an inverse correlation with hospitalization frequency. Despite the modest effect size, research indicates that mothers exhibiting superior practices may mitigate disease episodes necessitating hospital admission, possibly by preventative measures such as vitamin D prophylaxis, infection control, and prompt medical intervention. Other research has identified analogous correlations between maternal caregiving activities and diminished child morbidity^41^.

Nevertheless, BMI exhibited no substantial correlation, potentially attributable to its susceptibility to undernutrition (micronutrient insufficiency). The BMI of young children may not adequately reflect nuanced differences in health habits when compared to direct measurements of height and weight. This signifies the necessity for careful interpretation and the use of more refined nutritional indicators (e.g., weight-for-age or height-for-age z-scores) to more accurately represent the impact of maternal practices. These findings underscore that knowledge alone is inadequate without successful implementation in practice. Intervention programs should focus enhancing maternal knowledge while also equipping mothers with practical skills, resources, and supportive environments to consistently use that knowledge in everyday caregiving.

The current study demonstrated a moderate positive association between mothers’ practice and knowledge ratings. This outcome suggests that mothers with superior awareness of vitamin D are likely to exhibit improved practices about its use and deficiency prevention, and vice versa. The correlation coefficient indicates a moderate positive association, suggesting that although knowledge and practice are interconnected, the relationship is not particularly robust; additional factors, such as cultural beliefs, socioeconomic status, or availability of vitamin D sources, may also impact maternal practices. The result is statistically significant (*p* < 0.001), indicating that this association is unlikely to be coincidental. This outcome aligned with Al-Ghraibawi et al. (2019), who discovered that an augmentation in total knowledge correlated with a rise in the overall practice score (*p* = 0.03). Contrary to the assertions of Abbas et al.^42^, who claimed that no correlation exists between mothers’ knowledge and practice.

Although the positive correlation between maternal knowledge, practices, and educational level has been reported in previous studies, the present findings provide important context-specific insights. A notable observation is the discrepancy between knowledge and practice, where a considerable proportion of mothers demonstrated satisfactory awareness but inadequate implementation in daily practices. This highlights that knowledge alone is insufficient to ensure optimal child health outcomes, and that behavioral, cultural, and environmental factors may play a critical role. These findings underscore the need for interventions that focus not only on increasing awareness but also on improving practical application and behavioral change among mothers.

As regards the relationship between mothers’ characteristics with their total practice scores regarding VDD, our study discovered that maternal age showed a statistically significant association with practice scores regarding VDD, indicating that practice scores varied across different age groups. This suggests that age may influence how mothers apply health knowledge into daily practices, possibly due to accumulated life experience, exposure to health information, or differences in health-seeking behavior. Younger mothers may have greater access to online resources and updated health education, while older mothers may rely more on traditional practices, which can either positively or negatively affect their performance in preventive care.

In addition, maternal educational level also demonstrated a positive association with practice scores. Educated mothers tend to better understand the importance of vitamin D in child growth and bone health and are more likely to adopt recommended preventive practices such as providing balanced nutrition, ensuring adequate sun exposure, and adhering to supplementation guidelines when necessary. This aligns with results showing that practice score was significantly associated with levels of education^22^. Additionally, another study concluded that significant differences were found between mothers’ practices in relation to education and income^35^. Also, Shakir et al.^43^ stated that there is a significant relationship between mother’s awareness about vitamin D deficiency and their demographic data such as level of education, mother occupation and type of family. On the contrary, Al-Ghraibawi et al.^44^ found that there is no statistical association between age, education, and the mean practice score.

## Conclusion

This study showed that maternal practices regarding vitamin D were more strongly associated with children’s health outcomes than awareness alone. Although most mothers demonstrated satisfactory awareness, more than half reported unsatisfactory practices, indicating a clear gap between knowledge and implementation. Better maternal practices were significantly associated with absence of vitamin D deficiency, earlier eruption of primary teeth, greater child weight and height, and fewer hospitalizations. Maternal age and educational level were also significantly associated with practice scores. These findings highlight the need for nursing-led interventions that focus not only on improving mothers’ awareness but also on supporting practical behavior change related to vitamin D supplementation, safe sun exposure, and dietary practices.

### Implication of practice

The study findings highlight the need for interventions that go beyond information sharing to include skill-building, behavioral support, barrier reduction and awareness-raising to actively support and sustain beneficial maternal practices in nutrition and preventive care. Researchers recommend pediatric nurses take a stronger role in continuous education for mothers during pregnancy and post-natal visits, focusing on vitamin D, deficiency risks and prevention. Suggested strategies include role modeling, workshops, follow-up support, awareness campaigns, and distribution of educational booklets. Routine reminders (verbal, email, text) and postnatal vitamin D supplementation for all newborns with insurance coverage are also advised. So, pediatric nurses have a pillar role in enhancing child health through teaching, counseling, and empowering mothers to adopt evidence-based behaviors that promote optimal growth and prevent nutrition-related diseases. Finally, further research should assess mothers’ knowledge before and after educational programs and explore factors influencing their awareness and practices.

### Limitations

This study has limitations that must be considered when assessing the results. Only relationships may be drawn between mother awareness and children’s health outcomes due to the cross-sectional approach. The study used convenience sampling from three Egyptian governorates, which may limit its applicability to other populations or contexts with different sociodemographic characteristics. Self-reported data on mothers’ knowledge and behaviour may be affected by recall bias and social desirability. Fourth, cultural practices, seasonal sun exposure, and economic barriers to supplements were not examined despite their potential effects on mother behavior and child health. Future studies should employ longitudinal designs, biochemical evaluations, and diverse geographic and cultural settings to better understand how maternal understanding and activities affect child health outcomes.

In addition, the study did not assess important determinants of vitamin D status such as dietary intake of vitamin D-rich foods, duration and pattern of sun exposure, and other environmental or lifestyle factors. These variables may have a significant influence on children’s vitamin D status and health outcomes. Future studies are recommended to include these factors to provide a more comprehensive understanding of the determinants of vitamin D deficiency.

## Data Availability

All data available in the article.
